# Systematic analysis of direct antiglobulin test results in post-artesunate delayed haemolysis

**DOI:** 10.1186/s12936-021-03735-w

**Published:** 2021-04-29

**Authors:** Tommaso Ascoli Bartoli, Luciana Lepore, Alessandra D’Abramo, Giovanna Adamo, Angela Corpolongo, Laura Scorzolini, Maria Letizia Giancola, Nazario Bevilacqua, Claudia Palazzolo, Andrea Mariano, Giuseppe Ippolito, Pierre Buffet, Emanuele Nicastri

**Affiliations:** 1 Lazzaro Spallanzani, National Institute for Infectious Diseases-IRCCS, Via Portuense, 292, Cap 00149 Rome, Italy; 2 UMRS 1134, Inserm, Université de Paris, 75015 Paris, France; 3 Laboratory of Excellence GREx, 75015 Paris, France

**Keywords:** Malaria, Artemisinins, Artesunate, Hemolytic anemia, Coombs test, Drug-related side effects and adverse reactions

## Abstract

**Background:**

Post-artesunate delayed haemolysis (PADH) is common after severe malaria episodes. PADH is related to the “pitting” phenomenon and the synchronous delayed clearance of once-infected erythrocytes, initially spared during treatment. However, direct antiglobulin test (DAT) positivity has been reported in several PADH cases, suggesting a contribution of immune-mediated erythrocyte clearance. The aim of the present study was to compare clinical features of cases presenting a positive or negative DAT.

**Methods:**

Articles reporting clinical data of patients diagnosed with PADH, for whom DAT had been performed, were collected from PubMed database. Data retrieved from single patients were extracted and univariate analysis was performed in order to identify features potentially related to DAT results and steroids use.

**Results:**

Twenty-two studies reporting 39 PADH cases were included: median baseline parasitaemia was 20.8% (IQR: 11.2–30) and DAT was positive in 17 cases (45.5%). Compared to DAT-negative individuals, DAT-positive patients were older (49.5 vs 31; p = 0.01), had a higher baseline parasitaemia (27% vs 17%; p = 0.03) and were more commonly treated with systemic steroids (11 vs 3 patients, p = 0.002). Depth and kinetics of delayed anaemia were not associated with DAT positivity.

**Conclusions:**

In this case series, almost half of the patients affected by PADH had a positive DAT. An obvious difference between the clinical courses of patients presenting with a positive or negative DAT was lacking. This observation suggests that DAT result may not be indicative of a pathogenic role of anti-erythrocytes antibodies in patients affected by PADH, but it may be rather a marker of immune activation.

**Supplementary Information:**

The online version contains supplementary material available at 10.1186/s12936-021-03735-w.

## Background

Severe malaria is a medical emergency which caused 409,000 globally estimated deaths in 2019 [1]. The World Health Organization (WHO) guidelines recommend the use of intravenous (iv) artesunate to treat severe malaria either in adults and children [2], considering both efficacy and short-term safety reported in two controlled trials in Asia and Africa [3, 4]. Nevertheless, only limited evidence is available concerning the safety of iv artesunate beyond the initial treatment period because trial designs and lack of infrastructures in endemic countries did not allow for a long-lasting follow-up. Recently, several reports described the occurrence of a late-onset haemolytic syndrome, specifically related to the use of artesunate, defined post-artesunate delayed haemolysis (PADH). It usually occurs more than 1 week after the iv artesunate course, in approximately 10–40% of hyperparasitaemic non-immune travellers [5, 6].

PADH is a non-recurring highly expected event in non-immune hyperparasitaemic patients after artesunate administration [7]. In 2011, 6 out of 25 patients (24%) included in a severe malaria European cohort, developed PADH with a median onset of 15.5 days (IQR 15–32) after the start of intravenous artesunate. Five of them presented with severe anaemia requiring blood transfusions (83.3%), all resulting in a full recovery [8]. Furthermore, other authors registered a similar PADH occurrence rate. Kurth et al*.* reported a 27.1% incidence of PADH in a cohort of 70 patients with severe malaria managed in European countries [9]. Three patients (15.8%) with PADH received blood transfusions, and two (10.5%) needed to be re-hospitalized (for 3 and 5 days, respectively). Jauréguiberry et al*.* described a French cohort of 123 imported severe malaria cases, in which 27% of patients experienced PADH after a successful antimalarial treatment [6]. The 85% of the PADH recorded were mild and only one patient required blood transfusion. Lastly, few PADH cases after oral artemisinin-based combination therapy (ACT) have been already reported [5, 10, 11].

Although not fully elucidated, PADH pathophysiology may be explained by the peculiar pharmacological effect of artemisinin derivatives. The rapid anti-malarial action of these drugs is related to “pitting”, a process whereby artemisinin-exposed parasites are removed from their host erythrocytes, within the spleen microcirculation [12–14]. After being “pitted”, once-infected erythrocytes re-enter the systemic circulation, but with a reduced lifespan [13, 15–17]. Thus, although “pitting” initially spares parasite-hosting erythrocytes, this positive effect may not be sustained [15]. The delayed synchronous clearance of once-infected erythrocytes would explain major features of PADH, such as the late onset and the occurrence in parasite-free patients. Despite the evidence supporting “pitting” phenomenon as a leading factor in PADH pathogenesis, an increasing number of cases presenting with positive direct antiglobulin test (DAT) and/or in whom anaemia resolved following administration of systemic steroids has been reported [10, 18–24]. This finding suggests a possible role of drug-induced antibody-mediated haemolysis contributing at least to some of the PADH cases [25].

DAT is a method used to demonstrate the presence of antibodies or complement fractions bound to the red blood cells (RBC) membrane. The test is performed using anti-human globulins that cause, in case of a positive result, a visible agglutination reaction [17]. Different reagents can be used to elicit RBC agglutination: a polyspecific one recognizing both IgG and C3d and two monospecific agents recognizing only one of the two molecules. DAT positivity in the context of haemolysis usually defines auto-immune haemolytic anaemia (AIHA) [17]. Different subtypes of AIHA can generally be classified according to DAT result: warm AIHA (wAIHA) is usually the result of polyclonal IgG binding on RBC surface, while cold AIHA (cAIHA) is related to the effect of clonal or oligoclonal IgM with further activation of the complement cascade, this latter recognized from C3d fraction bound to RBC [26]. A mixed form, characterized by the recognition of both IgG and C3d on RBC membrane, has also been described. Noteworthy, a positive DAT alone does not allow for AIHA diagnosis. The test indeed, can be positive in 0.1% of healthy donors and positivity prevalence can rise up to 1–15% in hospitalized patients affected by acute illnesses, even in absence of haemolysis [17, 26]. Moreover, malaria and other systemic infections can be associated with a positive DAT, but the true role of immune mechanisms in contributing to malarial anaemia is not easy to determine [27, 28]. Finally, AIHA can be idiopathic or secondary to drugs exposure, autoimmune or lymphoproliferative disorders.

The aim of this systematic review was to collect demographic, clinical, and haematological data of all published PADH cases in which DAT results were reported and to compare the main clinical features of patients presenting with a positive or negative result. An additional objective was to investigate therapeutic strategies currently used for PADH management.

## Methods

**Fig. 1:**
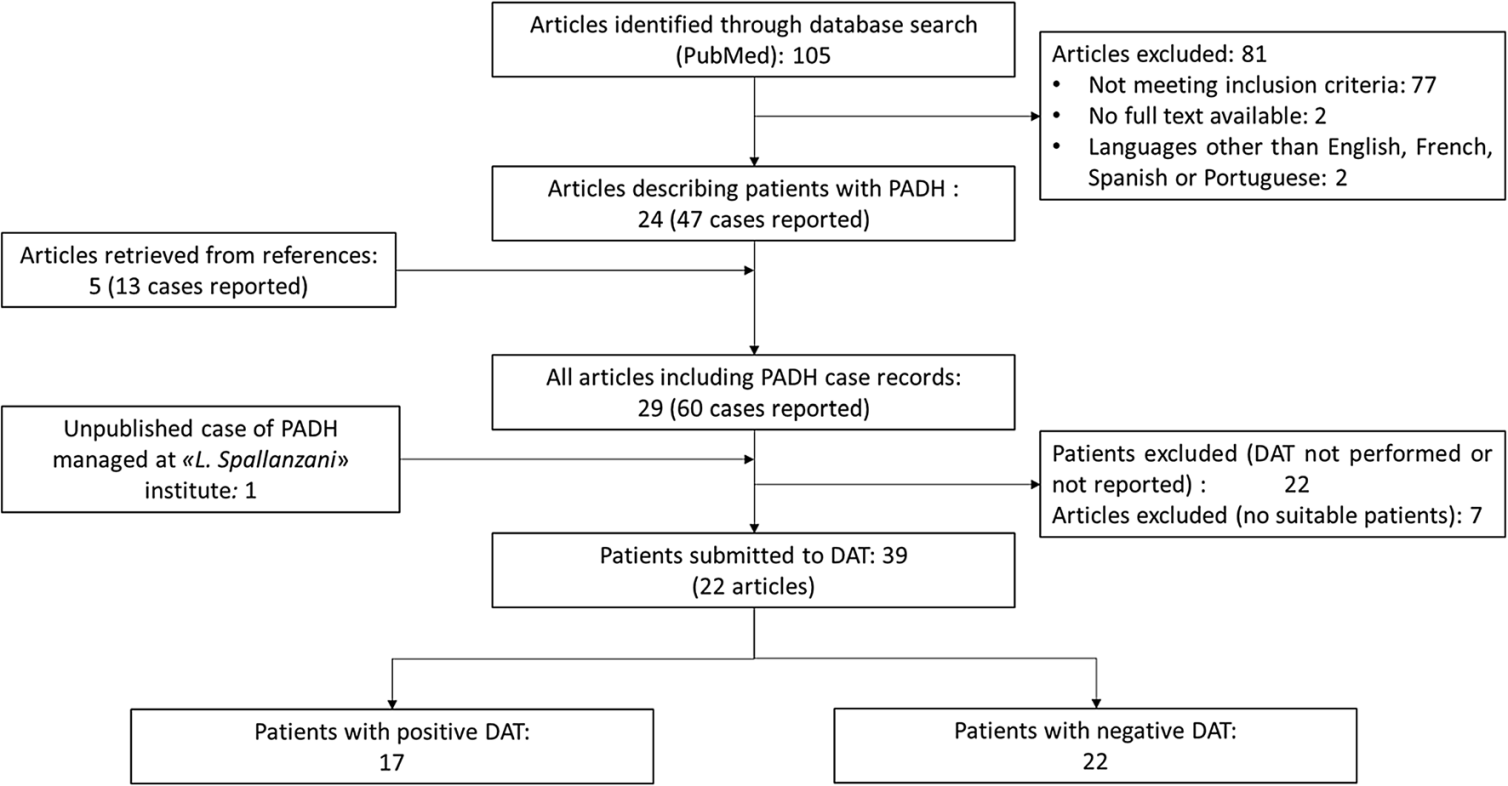
Systematic analysis flowchart. Reports of patients affected by PADH with an available DAT result

“Preferred Reporting Items for Systematic Reviews and Meta-Analysis” (PRISMA) recommendations were followed in the process of conducting this systematic analysis [29]. All published records of patients affected by PADH with a reported DAT result were included in the analysis. PADH was defined as a drop > 10% of the haemoglobin level occurred after ≥ 7 days since the start of artemisinin-based anti-malarial therapy, in the context of haemolysis laboratory signs not related to malaria recrudescence: an increase > 10% of the LDH blood concentration and/or LDH plasma level > 390 UI/l, or a plasma haptoglobin level < 0.1 g/l [6]. Severe malaria was defined according to WHO criteria [2]. As PADH has also been described after uncomplicated malaria episodes, these cases were considered suitable for the enrollment. Accordingly, patients were included in the analysis if they had received at least one dose of artemisinin derivatives, either as parenteral, oral, intramuscular or intrarectal formulations. PubMed database was investigated using a string of MeSH terms [("hemolysis" OR "anaemia, hemolytic") AND ("artemisinins" OR "artemisinine" OR "artesunate" OR "artemether" OR "artemether, lumefantrine drug combination" OR "dihydroartemisinin")] and all the papers published before October 31, 2019 were reviewed. All articles in English, French, Portuguese or Spanish language were included in the analysis. The reference list of all selected studies was investigated to identify other relevant articles. Titles and abstracts were independently screened by two authors. Data collected from every single patient were extracted from the selected studies by the same two authors and the final database was finally approved by the whole authors’ consensus. One clinical case included in the analysis is part of the severe malaria Italian multicentre study and has not yet been published before (Fig. 1).

Collected information included demographic data, travel history, clinical presentation, haematological, biochemical and parasitological tests, clinical management and follow-up. Comparisons between the groups of patients presenting with positive and negative DAT were performed using Mann–Whitney U-test, while Fisher’s exact test was used to analyse categorical variables. The same tests were used to assess differences in clinical variables between the group of patients who were treated with systemic steroids and those who were managed with clinical observation and RBC transfusions only. Statistical analysis was performed using STATA software.

## Results

Figure 1 shows the algorithm used to select the studies included in the review. Twenty-two articles reporting clinical features of 38 patients affected by PADH, who had been tested with DAT, were identified. Another unpublished case, managed at the National Centre for the Infectious Diseases “*L. Spallanzani*” and enrolled in the Italian multicentre malaria cohort, was also included in the analysis. Epidemiological, clinical, and therapeutic characteristics of the cohort patients are summarized in Additional file 1. Cumulative data are presented in Table 1 as aggregate case series and stratified according to positive (17/39 patients, 43.6%) or negative (22/39 patients, 56.4%) DAT result. DAT results were reported in detail in 13 out of the 17 positive patients (76.5%): five of them presented an IgG pattern (38.5%), six showed a C3d positivity (46.1%), while the remaining two cases were characterized by a mixed condition (15.4%).

**Table 1:** Epidemiological, clinical, and therapeutic characteristics of patients with PADH and at least one available DAT

Parameters	All patients (n = 39)	Patients with positive DAT (n = 17)	Patients with negative DAT (n = 22)	*p*
Age, median (IQR)	44 (24.5–51.5)	49.5 (43.2–54.7)	31 (20.5–48)	0.01
Female sex, number of patients (%)	13/39 (33.3%)	4/17 (23.5%)	9/22 (40.9%)	0.3
Born in malaria endemic countries, number of patients (%)	4/26 (15.4%)	1/10 (10%)	3/16 (18.7%)	1
Resident in malaria endemic countries, number of patients (%)	7/28 (25%)	2/11 (18.2%)	5/17 (29.4%)	0.7
*P. falciparum*, number of patients (%)	35/39 (89.7%)	15/17 (88.2%)	20/22 (90.9%)	1
*P. falciparum* + *P. vivax*, number of patients (%)	3/39 (7.7%)	2/17 (11.8%)	1/22 (4.5%)	0.6
*P. vivax*, number of patients (%)	1/39 (2.6%)	0/17 (0)	1/22 (4.5%)	1
Parasitaemia, median (IQR)	20.8% (11.2–30)	27% (21–34)	17% (9–22)	0.03
Hyperparasitaemia (> 10%), number of patients (%)	28/36 (77.8%)	14/17 (82.3%)	14/19 (73.7%)	0.7
Number of WHO severe malaria criteria [2], median (IQR)	3 (2–3)	3 (2–4)	3 (2–3)	0.6
Patients with acute kidney injury, number of patients (%)	16/35 (45.7%)	10/16 (62.5%)	6/19 (31.6%)	0.09
Parasite clearance time in days, median (IQR)	3 (2–4)	4 (3–5.5)	3 (2–4)	0.3
Intravenous artesunate, number of patients (%)	32/39 (82%)	15/17 (88.2%)	17/22 (77.3%)	0.4
Intrarectal artesunate, number of patients (%)	2/39 (5.1%)	1/17 (5.9%)	1/22 (4.5%)	1
Artesunate + oral ACT, number of patients (%)	17/37 (45.9%)	7/15 (46.7%)	10/22 (45.4%)	1
Not receiving artesunate (only other artemisinines), number of patients (%)	5/39 (12.8%)	1/17 (5.9%)	4/22 (18.2%)	0.4
Oral quinine, number of patients (%)	10/37 (27%)	6/15 (40%)	4/22 (18.2%)	0.3
Oral doxycycline, number of patients (%)	10/37 (27%)	5/15 (33.3%)	5/22 (22.7%)	0.7
Oral clindamycin, number of patients (%)	4/37 (10.8%)	0/15 (0)	4/22 (18.2%)	0.1
Days of intravenous Artesunate therapy, median (IQR)	3 (2–4)	3 (2–3.7)	3 (2.7–4)	0.8
Basal haemoglobin (Hb), median (IQR)	12.4 (10.7–13.8)	12.5 (11.2–13.2)	11.8 (10.5–14.3)	0.8
Time to PADH onset, in days, median (IQR)	12 (8.5–14)	13 (8.2–14)	11 (9–13)	0.5
Time to Hb Nadir during PADH, in days, median (IQR)	14 (11–15)	14.5 (12.2–16.7)	13 (11.2–15)	0.3
Hb level at nadir, median (IQR)	6 (5.2–6.8)	6.5 (5.4–6.9)	5.9 (4.8–6.4)	0.2
IgG DAT, number of patients (%)	–	5/13 (38.5%)	–	–
C3d DAT, number of patients (%)	–	6/13 (46.1%)	–	–
IgG/C3d DAT, number of patients (%)	–	2/13 (15.4%)	–	–
Patients treated with systemic corticosteroids, number of patients (%)	14/37 (37.8%)	11/16 (68.7%)	3/21 (14.3%)	0.002
Patients receiving transfusions, number of patients (%)	29/39 (74.4%)	12/17 (70.6%)	17/22 (77.3%)	0.7
Number of red blood cells units transfused, median (IQR)	2 (0–4)	2 (0–4)	3.5 (0–4)	0.5

Median age was 44 years (IQR 24.5–51.5): 49.5 years (IQR 43.2–54.7) and 31.0 years (IQR 20.5–48.0) in positive and negative DAT patients, respectively (p = 0.01). Overall, 35 malaria infections were sustained by *Plasmodium falciparum* (89.7%) and one by *Plasmodium vivax* (2.6%), while the remaining three patients were affected by a *P. falciparum*/*P. vivax* mixed infection (7.7%). The median baseline parasitaemia was 20.8% (IQR 11.2–30); parasite count was higher in DAT positive comparing to DAT negative PADH patients [27% (IQR 21–34) versus 17% (IQR 9.0–22.0) respectively (p = 0.03)]. Patients with positive DAT had a not statistically significant higher prevalence of previous acute kidney injury (AKI) (p = 0.09). Thirty-four patients (87.2%) had been treated with artesunate (32 intravenously and two intrarectally), usually combined with another antimalarial drug and/or followed by oral artemisinin-based combination therapy (ACT). Five patients (12.8%) had never received artesunate and experienced PADH after being treated with other artemisinin derivatives combined with different classes of anti-malarials.

Median time between antimalarial treatment start and PADH onset was 12 days (IQR 9–14), while median time to reach the haemoglobin nadir was 14 days (IQR 11–15). Median nadir was 6.0 g/dl (IQR 5.2–6.8) and blood transfusions were prescribed to 29 patients (74.4%) using a median number of two RBC units considering the whole PADH cohort. No significant differences were observed between patients presenting with a positive or negative DAT in terms of clinical features and outcome. Median haemoglobin level at nadir was 6.5 g/dl in DAT-positive (IQR 5.4–6.9) and 5.9 g/dl in DAT-negative patients (IQR 4.8–6.4), respectively (p = 0.2), while median time to nadir was 14.5 days (IQR 12.2–16.7) in DAT-positive and 13 (IQR 11.2–15) in DAT-negative patients, respectively (p = 0.3). Furthermore, the median amount of packed erythrocytes needed for transfusion support was similar between the two groups: two (IQR 0–4) and 3.5 (IQR 0–4) transfused RBC units in DAT-positive and DAT-negative patients, respectively (p = 0.5).

The lack of obvious differences between DAT negative and DAT positive patients in terms of time to onset, time to Hb nadir and Hb concentration at nadir suggests that antibodies causing DAT positivity may not be major operators of delayed anaemia. However, data regarding clinical course and outcomes should be interpreted cautiously, as they could have been influenced by the heterogeneous therapeutic strategies adopted.

Data concerning PADH pharmacological management were available for 37 patients; of them, 14 patients (37.8%) received either oral or intravenous steroids (Additional file 2). Systemic steroids were prescribed to 11/16 DAT-positive patients (68.7%) and to 3/21 DAT-negative patients (14.3%), respectively (p = 0.002) (Table 1). All the cases described resulted in a complete clinical recovery and no deaths were reported. However, the outcomes description and the follow-up duration were quite heterogeneous between the articles included.

## Discussion

Thirty-nine PADH case reports have been identified, in which clinical and immune haematological characteristics of patients, including DAT, were described. A previous review of 19 PADH cases reported worldwide in 2010–2012, has been published in 2013 [30]. The cohort reported here expands the former case series. Furthermore, a detailed analysis of clinical presentation, characteristics and therapeutic strategies is provided, with a separate description of patients presenting either with a positive or negative DAT. PADH has attracted clinical attention because it often occurs well after resolution of malaria-related symptoms and after complete parasite clearance and is neither directly related to active infection nor to the presence of parasites [8, 9]. Currently, weekly follow-up visits up to one month following artemisinin derivatives treatment are strongly recommended to detect and manage this condition, mainly in case of both high baseline parasitaemia and intravenous artesunate treatment [16]. In confirmed PADH cases, clinical tests performed to exclude common haemolytic disorders, immunologic diseases or drug-induced haemolysis, such as glucose-6-phosphate dehydrogenase deficiency, usually give inconclusive results [8, 9].

Interestingly, 17 (43.6%) of the patients included in the analysis presented a positive DAT result, either with an IgG, C3d or mixed pattern. The observation of a positive DAT in almost half of the PADH patients reported in the available literature, may suggest the possibility of different mechanisms underlying late-onset haemolysis in malaria patients. This finding may indicate the need for an active diagnostic attitude aimed at excluding a possible AIHA, in every case meeting PADH diagnostic criteria. This approach would help to early identify patients who could benefit from AIHA-specific treatment strategies, such as systemic steroid administration.

Moreover, no significant clinical differences were observed between the groups of patients presenting with a positive or negative DAT. Most of the patients included in the review showed a similar clinical course, with a median time to PADH onset occurring 12 days after artemisinin derivatives administration start, a low Hb nadir (6 g/dl) reached at day 14, a high need for RBC transfusion (74% of the patients) and a positive outcome. This observation suggests that, despite different DAT results, pathophysiologic mechanisms underlying the haemolytic process may be similar for all included patients and not related to DAT positivity. According to the latter hypothesis, the finding of a positive DAT may be only a nonspecific marker of systemic immune activation, triggered by the recent malarial episode, instead of a real determinant of the haemolytic anaemia [31].

Several factors can contribute to anaemia in patients affected by malaria, including acute intravascular haemolysis related to mechanical rupture of both infected and uninfected RBC and extravascular retention and/or phagocytosis of erythrocytes during splenic circulation [32]. This latter might be induced by mechanical splenic retention of RBC that lost their deformability or by opsonization/phagocytosis related to the effect of immunoglobulins, complement cascade, low levels of CD55, membrane expression of ring-surface protein 2 (RSP-2) and Rhoptry associated protein 2 (RAP-2). A reduced deformability of both infected and uninfected RBC is a well-known feature of acute malaria and it was recognized as a predictor of anaemia in severe cases [33–35]. In fact, expression of *Plasmodium* proteins on RBC membrane and increase in oxidative stress induce profound alterations in size, robustness and deformability of the erythrocytes. Consequently, their rheological properties and capacity to flow through microvessels of the spleen and peripheral circulation are compromised with an increase of the haemolysis burden. Dyserythropoiesis, bone marrow failure and drug-related side effects are other relevant factors contributing to malarial anaemia. It might be difficult to distinguish PADH when superimposed on that pathophysiological background [36–44]. Moreover, artemisinins may cause haemolysis through several other unexplored mechanisms, such as the modification of cellular metabolic pathways, the increase in the oxidative stress or the possibility to act as a hapten, inducing an immune mediated process.

As a matter of fact, circulating anti-erythrocytes antibodies are commonly detected during malaria episodes and the role of Ig-mediated phagocytosis of both infected and uninfected RBCs has been recognized as a major determinant of malarial anaemia [25, 45, 46]. In most of the cases included in the present review, DAT was performed only at the time of PADH diagnosis and the result was reported as a dichotomous variable (*positive* vs *negative*). It is important to recognize that a positive result at the time of PADH diagnosis, may merely reflect the persistence of antibodies bound to *self* or *Plasmodium* antigens, abnormally exposed by once-infected RBC since the acute malaria phase, rather than the occurrence of newly produced, drug-induced anti-erythrocytes immune globulins.

Therefore, it would be useful, as a further step towards a better understanding of PADH, to perform a longitudinal prospective evaluation of haemolysis markers and DAT results in patients affected by severe malaria, in order to identify a possible link between a delayed rise in anti-erythrocytes autoantibodies activity and haemolysis occurrence. Furthermore, indirect antiglobulin test and serologic assays aimed at determining the antigenic targets of anti-erythrocytes antibodies, may add useful information to the comprehension of the clinical phenomenon.

Data concerning PADH long-term follow-up, time to recovery and outcomes are partially lacking in most of the included articles. This additional information could be useful to collect in order to uncover possible differences in clinical course of patients presenting with a positive or a negative DAT. Considering the relatively low number of severe malaria cases in non-endemic countries, the issue should be further evaluated in a multicentre prospective observational study. This methodology would help to define the real benefit of implementing a specific strategy for early diagnostic approach and best therapeutic options in patients with severe PADH in the context of a positive DAT.

The results obtained from the literature review show that use of systemic steroids in patients affected by PADH is mainly driven by clinical decision, usually supported by a positive DAT, despite limited clinical evidence. Currently, there are no guidelines for the treatment of PADH. Considering the lack of effective therapies, the possible severity of the anaemia and potential adverse effects of transfusion, clinicians may “empirically” prescribe corticosteroids, especially in DAT positive patients. Systemic steroids, mainly predniso(lo)ne, are widely used in primary and secondary AIHA [26]. When short courses are used, safety is generally not an issue. Variable regimens were used to treat PADH, and the level of evidence for efficacy is low in the absence of comparative trials.

This review presents a few limitations. First, several PADH published cases did not mention any DAT result. The choice to include only PADH cases in which DAT had been considered as a relevant diagnostic procedure, inevitably leads to a selection bias related to the possible exclusion of milder haemolytic episodes, in which AIHA had not been considered in the differential diagnosis and systemic steroids had not been evaluated as a suitable therapeutic option. Second, as discussed above, DAT results are highly variable among general population and are distributed over a broad range, therefore it would have been preferable to have the test performed by a single central laboratory and the result reported as a continuous non-binary variable.

Given the mentioned confounding factors, the issue should be best investigated through a targeted prospective study. Severe malaria patients at high risk for PADH should be recruited from different settings (endemic areas and tropical medicine wards in non-endemic countries) and blood and serum samples stored for the monitoring of multiple haemolysis markers, DAT and indirect antiglobulin test. Assessment of anti-erythrocytes antibodies specificity would also add useful information and a long-term follow-up should be scheduled in order to achieve a reliable clinical description.

## Conclusions

The present review points out that DAT positivity is a common finding among patients affected by PADH. Nevertheless, the analysis shows that DAT positivity is not associated with worse clinical features, laboratory parameters or outcomes and may be a nonspecific expression of malaria-related systemic immune activation. However, data included in the review were heterogeneously collected and information concerning PADH time to recovery and follow-up was lacking. Therefore, a prospective observational study is needed in order to understand if DAT positivity may identify a subpopulation of patients in which antibody-mediated haemolysis contributes to PADH features, eventually leading to a worse outcome and possibly requiring a targeted therapeutic approach.

## Supplementary Information

**Additional file 1.** Epidemiological, clinical, and therapeutic characteristics of patients with PADH and at least one available DAT result.

**Additional file 2.** Epidemiological, clinical, and therapeutic characteristics of patients with PADH with an available DAT result reported as aggregate case series and stratified according to the administration of systemic steroids.

## Data Availability

The datasets used and analysed during the current study are available from the corresponding author on reasonable request.

## Abbreviations

PADH: Post-artesunate delayed haemolysis
DAT: Direct antiglobulin test
iv: Intravenous
LDH: Lactate dehydrogenase
AKI: Acute kidney injury
ACT: Artemisinin-based combination therapy
RBC: Red blood cells
AIHA: Auto-immune haemolytic anaemia
wAIHA: Warm AIHA
cAIHA: Cold AIHA
Hb: Haemoglobin
