# Supplementary material for: Systematic analysis of direct antiglobulin test results in post-artesunate delayed haemolysis
Source: Malar J. 2021 Apr 29;20:206. doi: 10.1186/s12936-021-03735-w (PMC8082776; doi:10.1186/s12936-021-03735-w)
Supplement: Supplementary file 1 — Additional file 1. Epidemiological, clinical, and therapeutic characteristics of patients with PADH and at least one available DAT result. [file 12936_2021_3735_MOESM1_ESM.docx]

**Supplementary file 1:** Epidemiological, clinical, and therapeutic characteristics of patients with PADH and at least one available DAT result.

| **Patient #** | **Age**  **(years)** | **Sex** | **Place of birth** | **Country of residence** | **Country of infection** | **Infecting species** | **Parasitaemia (%)** | **Number of WHO Severity criteria [2]*** | **Kidney injury** | **Antimalarial therapy** | **AS duration**  **(days)** | **Parasite clearance time (days)** | **Basal Hb (g/dl)** | **PADH**  **onset (days)** | **Hb nadir (g/dl)** | **Peak LDH level (U/l)** | **DAT (+/-)** | **Treatment with Systemic**  **cortico- steroids** | **RBC**  **Transfusion (**)** | **Ref.** |
| --- | --- | --- | --- | --- | --- | --- | --- | --- | --- | --- | --- | --- | --- | --- | --- | --- | --- | --- | --- | --- |
| 1 | 44 | F | Spain | Spain | Senegal | Pf | 37 | 4 | Yes | AS, AP, DP | 3 | - | 8.7 | 12 | 5.6 | 1469 | + | No | Yes (3) | 22 |
| 2 | 29 | M | India | Angola | Angola | Pf | 17 | 3 | Yes | AS, AL | 3 | - | 11.9 | 13 | 8.3 | 1005 | - | No | No | 22 |
| 3 | 53 | M | - | - | - | Pf | 34 | 3 | No | AS, Q, AP | 1 | 4 | 12.9 | 20 | 6.9 | - | + (C3d) | No | No | 10 |
| 4 | 50 | F | - | - | - | Pf | 11 | 2 | No | AS, AP | 3 | 3 | 11.8 | 13 | 4.5 | - | - | No | Yes (4) | 10 |
| 5 | 44 | F | - | - | - | Pf | 37 | 1 | No | AS,Q, AL | 2 | 4 | 10 | 9 | 6.1 | - | + (IgG, C3d) | Yes | Yes (6) | 10 |
| 6 | 5 | M | - | - | - | Pf | 12 | 3 | No | AS, Q, AL | - | 4 | 12.9 | 8 | 6.1 | - | - | No | No | 10 |
| 7 | 50 | F | - | - | - | Pf | 30 | 2 | No | AS, AL | - | 10 | 11.6 | 13 | 6.9 | - | + (IgG, IgM) | Yes | Yes (2) | 10 |
| 8 | 71 | M | - | - | - | Pf | 20 | 6 | Yes | AS, Q, AL | - | 7 | 8.1 | 13 | 6 | - | - | No | Yes (24) | 10 |
| 9 | 17 | F | Ivory Coast | Ivory Coast | Ivory Coast | Pf | 0.8 | 2 | No | AS, AL | 2 | 3 | 12.6 | 8 | 4.6 | 658 | + (IgG; C3d) | Yes | No | 18 |
| 10 | 43 | M | - (Caucasian) | - (Europe) | Gabon | Pf | 30 | 3 | Yes | AS, AL | 4 | 4 | 12.3 | 8 | 5.3 | 3035 | + (C3d) | Yes | No | 19 |
| 11 | 78 | M | - | - | - | Pf | 27 | 2 | Yes | AS (others NR) | - | 1.5 | 13.2 | 15 | 6.4 | - | + | Yes | Yes (9) | 20 |
| 12 | 54 | M | Germany | Germany | The Gambia | Pf | 21 | 5 | Yes | AS, Dx, AP | 3 | 7 | - | 14 | 5.7 | 1000 | + (IgG anti-E) | No | Yes (2) | 21 |
| 13 | 55 | M | Germany | Germany | Senegal, The Gambia | Pf | 20 | 3 | Yes | AS, AP | 3 | - | 14.7 | 13 | 6.6 | 1500 | - | No | No | 21 |
| 14 | 50 | M | India | India | India | Pf/Pv | 45 | 5 | Yes | AS (others NR) | - | - | 12.5 | 8 | 4.1 | 2436 | + (IgG) | Yes | Yes (-) | 23 |
| 15 | 55 | M | - (Europe) | - (Europe) | Zambia, Malawi, Mozambique South Africa | Pf | 28.6 | 1 | No | AS, Q, Dx, AP | 1 | 4 | - | 14 | 5.1 | - | + (C3d) | Yes | Yes (4) | 24 |
| 16 | 55 | M | - (Europe) | - (Europe) | Sierra Leone | Pf | 8.7 | 2 | Yes | AS, Q, Dx | 3 | 7 | - | 20 | 9.6 | - | + (C3d) | No | No | 24 |
| 17 | 40 | M | - (Europe) | - (Europe) | Namibia, Botswana, Zimbabwe, Malawi, Mozambique | Pf | 45 | 3 | Yes | AS, Q, Dx | 4 | 3 | - | 13 | 6.7 | - | + (C3d) | Yes | Yes (1) | 24 |
| 18 | 49 | M | - (Europe) | - (Europe) | Liberia | Pf | 25 | 4 | Yes | AS, DP | 4 | 3 | - | 13 | 7.7 | - | + (C3d) | Yes | Yes (1) | 24 |
| 19 | 45 | M | Bangladesh | Bangladesh | Bangladesh | Pf | 13.8 | 2 | No | AS, AL | 2 | 7 | - | 14 | 4.9 | 887 | - | No | Yes (3) | 47 |
| 20 | 32 | M | - (Europe) | - (Europe) | Togo | Pf | - | - | - | AR im, DP | 0 | - | 12.8 | 11 | 4.1 | - | - | No | Yes (4) | 48 |
| 21 | 44 | M | Canada | Canada | Cameroon | Pf | 12.3 | 5 | Yes | AS, AP | 3 | 3 | 16.4 | 14 | 6.8 | - | + (IgG) | No | Yes (11) | 49 |
| 22 | 55 | M | Canada | Canada | DRC | Pf | 35 | 4 | No | AS, AP | 3 | 3 | 10.5 | 15 | 5.9 | 1015 | - | No | Yes (2) | 49 |
| 23 | 36 | F | - | Djibouti | Sudan/Djibouti | Pf | 30 | 3 | No | AS, DP | 2 | 7 | 14.3 | 11 | 5.7 | - | - | No | Yes (4) | 50 |
| 24 | 34 | M | Italy | Italy | Central African Republic | Pf | 6 | 3 | Yes | Q, Dx, AL | 0 | 3 | 7.4 | 10 | 5.9 | 2071 | - | No | Yes (4) | 51 |
| 25 | 30 | F | - (Europe) | - (Europe) | India | Pf | 30 | 1 | No | AS, Dx | 7 | 3 | 11.3 | 15 | 5.7 | 1437 | - | No | Yes (2) | 8 |
| 26 | 49 | M | - (Europe) | - (Europe) | Angola | Pf | 9 | 3 | Yes | AS, Dx, AL | 4 | 1.5 | 15.5 | 15 | 5.7 | 1489 | - | No | Yes (-) | 8 |
| 27 | 22 | F | Italy | Italy | Sierra Leone | Pf | 6.9 | 1 | No | AS, AL | 2 | 4 | 14.4 | 14 | 5.6 | 2406 | - | No | Yes (-) | 52 |
| 28 | 31 | M | Canada | Canada | South Sudan | Pf | 22 | 2 | No | AS, Dx, AP, AL | 3 | 1.5 | 14.4 | 8 | 6.8 | 3429 | - | No | Yes (8) | 53 |
| 29 | 6 | F | Spain | Spain | Senegal | Pf | 5 | - | - | AS, DP | 4 | 2 | - | 10 | 7.4 | 751 | - | Yes | No | 54 |
| 30 | 4 | F | Spain | Spain | Senegal | Pf | 15 | - | - | AS, DP | 3 | 3 | - | 10 | 6.3 | 1831 | - | Yes | Yes (1) | 54 |
| 31 | 6 | M | - | - | The Gambia | Pf/Pv | 25 | - | - | AS, AP, DP | 2 | 3 | - | 8 | 7.5 | 1831 | + | Yes | No | 54 |
| 32 | 27 | F | - | Ghana | Ghana | Pf | 7.5 | 3 | No | AS, Cl | 4 | 3 | 11.4 | 12 | 4.7 | 3994 | - | No | Yes (4) | 55 |
| 33 | 47 | F | Italy | Italy | Niger | Pf | 18 | 2 | No | AS, Dx, Cl | 3 | 2 | 11.6 | 8 | 6 | 8028 | - | No | Yes (8) | UC |
| 34 | 55 | M | Italy | Italy | Kenya | Pf | 6 | 1 | No | AL | 0 | 2 | 13.2 | 9 | 6.9 | 797 | + | Yes | Yes (4) | 11 |
| 35 | 0.5 | M | India | India | India | Pv | >2 | - | - | AS, Cl | - | - | 10.4 | 10 | 2.8 | 1051 | - | Yes | Yes (-) | 56 |
| 36 | 21 | M | Japan | Japan | India | Pf/Pv | - | 2 | No | AL | 0 | 2 | 8.5 | 8 | 3.8 | 840 | - | No | Yes (-) | 57 |
| 37 | 20 | M | Japan | Japan | Cameroon | Pf | 20.6 | 1 | No | AL | 0 | 4 | 14 | 13 | 7.2 | 1466 | - | No | No | 58 |
| 38 | - | F | - | - | Burkina Faso | Pf | 30 | - | Yes | AS ir, Q, Cl | 0 | - | - | - | - | - | - | - | Yes (4) | 5 |
| 39 | - | M | - | - | Burkina Faso | Pf | 25 | - | Yes | AS ir, Q, Dx | 0 | - | - | - | - | - | + (IgG) | - | Yes (2) | 5 |

**Abbreviations key**: Pf: P. falciparum. Pv: P. vivax. AS: intravenous artesunate. AS ir: intrarectal artesunate. AL: artemether/lumefantrine. DP: dihydroartemisinin/piperaquine. AR im: intramuscular artemether. AP: atovaquone/proguanil. Q: quinine. Dx: doxycycline. Cl: clindamycin. NR: not reported. PADH: post-artemisinin delayed haemolysis. DAT: direct antiglobulin test. RBC: red blood cells. UC: unpublished case.

*: on admission or during follow-up. **: Total amount of RBC units transfused (when reported by the authors).
