# Supplementary material for: Systematic analysis of direct antiglobulin test results in post-artesunate delayed haemolysis
Source: Malar J. 2021 Apr 29;20:206. doi: 10.1186/s12936-021-03735-w (PMC8082776; doi:10.1186/s12936-021-03735-w)
Supplement: Supplementary file 2 — Additional file 2. Epidemiological, clinical, and therapeutic characteristics of patients with PADH with an available DAT result reported as aggregate case series and stratified according to the administration of systemic steroids. [file 12936_2021_3735_MOESM2_ESM.docx]

**Additional file 2:** epidemiological, clinical, and therapeutic characteristics of patients with PADH with an available DAT result reported as aggregate case series and stratified according to the administration of systemic steroids.

| **Parameters** | **Patients treated with steroids**  **(n = 14)** | **Others (n = 23)** | ***p*** |
| --- | --- | --- | --- |
| **Age**, median (IQR) | 43.5 (6-51.2) | 44 (29-53) | *0.5* |
| **Female sex**, number of patients (%) | 5/14 (35.7%) | 7/23 (30.4%) | *0.7* |
| **Born in malaria endemic countries**, number of patients (%) | 2/9 (22.2%) | 2/17 (11.8%) | *0.6* |
| **Resident in malaria endemic countries**, number of patients (%) | 3/10 (30%) | 4/18 (22.2%) | *0.7* |
| ***P. falciparum***, number of patients (%) | 11/14 (78.6%) | 22/23 (95.6%) | *0.1* |
| ***P. falciparum* + *P. vivax***, number of patients  (%) | 2/14 (14.3%) | 1/23 (4.3%) | *0.5* |
| ***P. vivax***, number of patients (%) | 1/14 (7.1%) | 0/23 (0) | *0.4* |
| **Parasitaemia**, median (IQR) | 27 (10.5-33.5) | 18 (10-26) | *0.2* |
| **Hyperparasitaemia (>10%)**, number of patients (%) | 10/13 (76.9%) | 16/21 (76.2%) | *1* |
| **Number of WHO severe malaria criteria [2]**, median (IQR) | 2 (1-3.2) | 3 (2-3.2) | *0.3* |
| **Patients with acute kidney injury**, number of patients (%) | 5/11 (45.4%) | 9/22 (40.9%) | *1* |
| **Parasite clearance time in days**, median (IQR) | 3 (2-4) | 3 (3-6) | *0.4* |
| **Intravenous artesunate**, number of patients  (%) | 13/14 (92.9%) | 19/23 (82.6%) | *0.6* |
| **Artesunate + oral ACT**, number of patients (%) | 8/12 (66.7%) | 9/23 (39.1%) | *0.2* |
| **Not receiving artesunate (only other artemisinines),** number of patients (%) | 1/14 (7.1%) | 4/23 (17.4%) | *0.6* |
| **Oral quinine**, number of patients (%) | 3/12 (25%) | 5/23 (21.7%) | *1* |
| **Oral doxycycline**, number of patients (%) | 2/12 (16.7%) | 7/23 (30.4%) | *0.4* |
| **Oral clindamycin**, number of patients (%) | 1/12 (8.3%) | 2/23 (9%) | *1* |
| **Days of intravenous Artesunate therapy**, median (IQR) | 2.5 (1.7-4) | 3 (1.5-3) | *0.7* |
| **Basal haemoglobin (Hb)**, median (IQR) | 12.4 (10.7-13) | 12.3 (10.7-14.4) | *0.7* |
| **Time to PADH onset**, in days, median (IQR) | 10 (8-13) | 13 (10-14) | *0.1* |
| **Time to PADH Nadir in days**, median (IQR) | 13.5 (10-15.2) | 15 (12-15.5) | *0.3* |
| **Hb level at nadir**, median (IQR) | 6.3 (5-7) | 5.9 (5.6-6.8) | *0.5* |
| **Positive DAT**, number of patients (%) | 11/14 (78.6%) | 5/23 (21.7%) | *0.00*  *16* |
| **Patients receiving transfusions**, number of  patients (%) | 10/14 (71.4%) | 17/23 (73.9%) | *1* |
| **Number of red blood cells units transfused**, median (IQR) | 1 (0-4) | 3 (0-4) | *0.4* |
